# The Role of Periodontitis and Periodontal Bacteria in the Onset and Progression of Alzheimer’s Disease: A Systematic Review

**DOI:** 10.3390/jcm9020495

**Published:** 2020-02-11

**Authors:** Mario Dioguardi, Vito Crincoli, Luigi Laino, Mario Alovisi, Diego Sovereto, Filiberto Mastrangelo, Lucio Lo Russo, Lorenzo Lo Muzio

**Affiliations:** 1 Department of Clinical and Experimental Medicine, University of Foggia, Via Rovelli 50, 71122 Foggia, Italy; diego_sovereto.546709@unifg.it (D.S.); filiberto.mastrangelo@unifg.it (F.M.); lucio.lorusso@unifg.it (L.L.R.);; 2 Department of Basic Medical Sciences, Neurosciences and Sensory Organs, Division of Complex Operating Unit of Dentistry, “Aldo Moro” University of Bari, Piazza G. Cesare 11, 70124 Bari, Italy; vito.crincoli@uniba.it; 3 Multidisciplinary Department of Medical-Surgical and Odontostomatological Specialties, University of Campania “Luigi Vanvitelli”, 80121 Naples, Italy; luigi.laino@unicampania.it; 4 Department of Surgical Sciences, Dental School, University of Turin, 10126 Turin, Italy

**Keywords:** Alzheimer’s disease, periodontitis, *porphyromonas gingivalis*

## Abstract

The evidence of a connection between the peripheral inflammatory processes and neurodegenerative diseases of the central nervous system is becoming more apparent. This review of the related literature highlights the most recent clinical, epidemiological, and in vitro studies trying to investigate possible connections between periodontal bacteria and the onset and progression of Alzheimer’s disease. This review was conducted by searching databases such as PubMed and Scopus using keywords or combinations such as Alzheimer’s Disease AND periodontal or dementia AND periodontitis OR periodontal. After eliminating overlaps and screening the articles not related to these issues, we identified 1088 records and proceeded to the selection of articles for an evaluation of the associative assumptions. The hypothesis suggested by the authors and confirmed by the literature is that the bacterial load and the inflammatory process linked to periodontal disease can intensify inflammation at the level of the central nervous system, favoring the occurrence of the disease. The analysis of the literature highlights how periodontal disease can directly contribute to the peripheral inflammatory environment by the introduction of periodontal or indirect pathogenic bacteria and proinflammatory cytokines locally produced at the periodontal level following bacterial colonization of periodontal defects.

## 1. Introduction

Pathologies related to mature adults in Western countries tend to be more frequent, with a heavier social and medical impact, taking into consideration the increasing average life expectancy [1]. Mature adults should be able to face old age in the best possible way, including reducing the most invalidating aspects of neurodegenerative pathologies, such as Alzheimer’s disease-associated dementia [2]. Among various problems, there is a reduced chewing ability associated with tooth loss causing malnutrition, whose main cause is represented by the periodontal disease [3]. Local inflammatory diseases, such as periodontal disease, represent a general and oral health problem in the elderly. The loss of dental elements leads to a reduction in their masticatory capacity. This in turn leads to undernourishment and, moreover, the presence of a local chronic inflammation that can influence pre-existing systemic pathologies, favoring their onset and progression [4]. Several studies have shown the possible correlation between periodontal disease and the onset and progression of Alzheimer’s disease, with an increasingly predominant role of inflammation and bacteria associated with periodontitis.

The most recent study on the subject is a case control study conducted by Holmer in 2019, which reports a correlation between periodontitis and early cognitive impairment and Alzheimer’s disease [5]. In addition, another study conducted by Díaz-Zúñiga in 2019 posed the question of the direct role of the *Aggregatibacter actinomycetemcomitans* bacterium, reaching the conclusion that the latter activates proinflammatory cytokine secretion by microglia [6].

In light of these findings, the question posed by the authors is how can periodontal disease effect the onset and progression of Alzheimer’s disease? Understanding these mechanisms can alert healthcare professionals and the dentists involved in the treatment of Alzheimer’s patients to the potential neurodegenerative effects of local inflammatory and infectious processes such as periodontal disease, allowing them to act preventively to treat the causes. Our hypothesis is that the inflammatory process related to the periodontal disease may influence Alzheimer’s disease pathogenesis by worsening dementia, its main manifestation, in a synergic way [7].

## 2. Materials and Methods 

The following systematic review was conducted based on the indications of the Prisma protocol.

The study was constructed on the PICO question: Population (patients with Alzheimer’s disease), Intervention (periodontal disease and periodontal bacteria), Control (patients who do not suffer from Alzheimer’s disease), and Outcome (the role of periodontal bacteria and inflammation induced by periodontal disease in the onset and progression of Alzheimer’s disease).

The formulation of the PICO question was as follows; what is the etiopathogenetic role of periodontal disease and periodontal bacteria in the onset and progression of Alzheimer’s disease compared to unaffected patients?

After an initial selection phase of the records identified from the databases, the potentially eligible articles were qualitatively evaluated to investigate the role of periodontal disease bacteria (*Porphyromonas gingivalis*, *Aggregatibacter actinomycetemcomitans*, *Fusobacterium nucleatum*, and *Treponema denticola*).

### 2.1. Eligibility Criteria

The works taken into consideration were literature reviews, in vitro experiments, and clinical studies concerning the role of periodontal disease and bacteria in the onset and progression of Alzheimer’s disease; we particularly considered articles investigating the role of bacteria such as *Porphyromonas gingivalis*, *Aggregatibacter actinomycetemcomitans*, *Fusobacterium nucleatum*, and *Treponema denticola* in association with Alzheimer’s disease that were conducted recently and published in English.

We focused on articles from the last 30 years because the investigation of a possible association between periodontal disease and Alzheimer’s disease has only been undertaken in the last 20 years.

The articles considered to be potentially eligible were those reporting on the association between periodontal disease and Alzheimer’s disease, excluding articles published more than 30 years ago and that did not present an abstract in English. The potentially eligible articles were finally subjected to a full text analysis to verify their use for qualitative analysis.

The inclusion and exclusion criteria applied in the full text analysis were the following.

- To include all those studies describing the association between Alzheimer’s disease and periodontal disease.
- To include all articles describing the role of bacteria such as *Porphyromonas gingivalis, Aggregatibacter actinomycetemcomitans, Fusobacterium nucleatum* and *Treponema denticola* in the onset and progression of the Alzheimer’s disease.
- To exclude all studies that were not written in English and were published before 1990.
- To exclude non-systematic literature reviews.

### 2.2. Research Methodology

Studies have been identified through bibliographic research on electronic databases.

The literature search was conducted on the search engines “PubMed” and “Scopus” between 10.09.2019 and 02.10.2019 and the last search for a partial update of the literature was conducted on 02.11.2019.

The following search terms were used on PubMed and Scopus. “Alzheimer’s Disease” AND “periodontal” (PubMed: 96, Scopus: 234); “Alzheimer’s Disease” AND “periodontitis“ (PubMed: 87, Scopus: 182); “dementia” AND “periodontal” (PubMed: 118, Scopus: 217); “Alzheimer’s Disease” AND “actinomycetemcomitans” (PubMed: 3, Scopus: 8); “Alzheimer’s Disease” AND “gingivalis “ (PubMed: 34, Scopus: 64); “Alzheimer’s Disease” AND “denticola” (PubMed: 9, Scopus: 20); “Alzheimer’s Disease” AND “nucleatum” (PubMed: 4, Scopus: 12) (Table 1).

### 2.3. Screening Methodology

The records obtained were subsequently examined by two independent reviewers (M.D. and D.S.) and a third reviewer (L.L.) acted as a decision-maker in situations of doubt. The screening included the analysis of the title and the abstract to eliminate the records not related to the topics of the review. Specifically, the identification of the articles related to the secondary outcome topics was made for each key word analyzing the title; the abstract; and, in doubtful cases, the full text. The various articles, selected from each keyword, were grouped and subject to overlap removal. The choice to manually and subsequently remove only the screening phase was made to facilitate the manual removal procedure.

After the screening phase, the overlaps were removed, and the complete texts of the articles were analyzed to identify those eligible for qualitative analysis. The results sought by the two reviewers were as follows.

(1) Primary outcome: associations between periodontitis and Alzheimer’s disease.
(2) Secondary outcome: associations between bacteria involved in the pathogenesis of periodontal disease and Alzheimer’s disease.

The fourth reviewer, with supervisory duties, was L.Lo.M. The K agreement between the two screening reviewers was 0.754 (Table 2). The K agreement was based on the formulas of the Cochrane Handbook for systematic reviews [8].

## 3. Results

A total of 1088 records were identified on the PubMed and Scopus databases (Table 1). After limiting the year of publication to between 1989 and 2019, there were 1082 records. With the application of the eligibility criteria (studies dealing with the topic of Alzheimer’s disease in relation to oral inflammatory processes and bacteria), there were 444 articles. There were 114 articles that discussed the role of periodontal bacteria in the onset and progression of Alzheimer’s disease; there were 36 articles after eliminating overlaps.

After applying the inclusion and exclusion criteria, the result was a total of 15 articles for the qualitative analysis.

**Figure 1:**
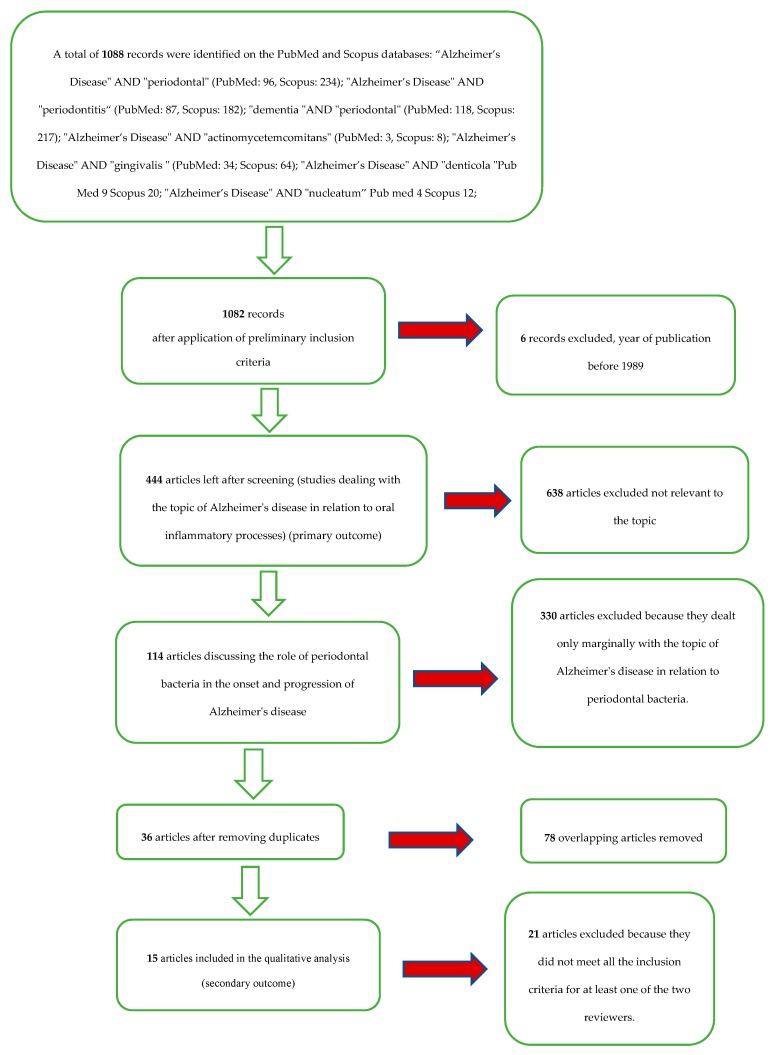
Flowchart of the different phases of the systematic review.

The selection and screening procedures are described in the flow chart in Figure 1.

### Study Characteristics and Data Extraction

The studies included for qualitative analysis were as follows.

- First outcome: The articles included for the first outcome (associations between periodontitis and Alzheimer’s disease) after the elimination of overlaps were about a hundred. The articles were then studied to deepen and update the reviewers’ knowledge on the subject of the association between Alzheimer’s disease and periodontitis. Articles were used to draft the discussion on the correlations between the two diseases and their respective etiopathogenesis.
- Second outcome: the articles included for the second outcome (associations between bacteria involved in the pathogenesis of periodontal disease and Alzheimer’s disease) were Wu et al. 2017 [9], Hayashi et al. 2019 [10], Poole et al. 2013 [11], Carter et al. 2017 [12], Liu et al. 2017 [13], Laugish et al. 2018 [14], Ide et al. 2016 [15], Ishida et al. 2017 [16], Dominy et al. 2019 [17], Nie et al. 2019 [18], Diaz-Zuniga et al. 2019 [6], Sparks Stein et al. 2012 [19], Noble et al. 2014 [20], Carter et al. 2017 [21], and Kamer et al. 2009 [22].

The extracted data included the paper (author, data, and journal), the type of study (in vitro, on animals, clinical study, systematic review), type of bacteria investigated (*Porphyromonas gingivalis*, *Aggregatibacter actinomycetemcomitans*, *Fusobacterium nucleatum*, and *Treponema denticola*), genes, antibodies or antigens under study, and number of cases and controls.

The data extracted for the secondary outcomes are shown in Table 3.

## 4. Discussion

### 4.1. Periodontal disease

Periodontal diseases are a group of chronic diseases characterized by a bacterial etiology and an inflammatory pathogenesis, which have a remarkable diffusion in the general population and are the principal cause of tooth loss [23]. The etiology of periodontal disease, as demonstrated by many studies, is bacterial; moreover, the causative microorganisms accumulate on the hard surfaces of teeth and inside periodontal pockets, forming a complex biofilm defined as bacterial plaque [24]. 

The biofilm complexity is represented by the bacterial composition, but also by the existing interactions between the microorganisms themselves and between the microorganisms and their habitat; in fact, there are more than 700 bacterial species present in the bacterial plaque which exhibit different grades of pathogenesis towards the host [25]. Similarly, inside the oral cavity, different microenvironmental conditions can originate, influenced by the microbial composition and vice versa [26,27]. 

Plaque accumulation causes qualitative modifications of the microbial component, with an increase in the number and proportion of Gram-negative anaerobic bacteria, which have more significant relevance in the pathogenesis of periodontitis. In fact, among these species, there are some which are considered to be specific periodontal pathogens: *A. actinomycetemcomitans*, *P. gingivalis*, *T. forsythia*, *T. denticola*, *P. intermedia*, *F. nucleatum*, *C. rectus*, etc. [28]. 

The periodontal pathogens mentioned above have different pathogenicity and virulence features that are fundamental in periodontal disease pathogenesis. After the accumulation of plaque and bacterial products, the host responds with an inflammatory reaction, which is theoretically able to remove the pathogenic microorganisms. However, these reactions are not always able to remove the etiologic agent; they can become chronic, with the recruitment of different cellular population (such as granulocytes, macrophages, and lymphocytes) and the production of proinflammatory cytokines, causing tissue remodeling and periodontal destruction [29]. Tissue damage is predominantly caused by the proteases released by the host immune system, such as neutrophil collagenases, which degrade the connective tissue collagen to produce the necessary space for the inflammatory infiltration [30].

During gingivitis, the host is able to withstand microbes but, if periodontitis occurs, the existing equilibrium between the immune response and subgingival microbes gets altered, leading to uncontrolled inflammation characterized by the production of a high level of inflammatory intermediates, such as IL-1, IL-6, IL-17, and TNF-α and low levels of IL-10 inside periodontal tissues [31]. 

Proinflammatory cytokines, such as TNF-α, IL-1, INF-γ, and PGE2, can be highly concentrated in periodontal pocket tissues, representing a source from which these inflammatory intermediates can be released. For this reason, periodontitis represents a major risk factor for pathologies such as cardiovascular diseases [32], respiratory infections, diabetes, and kidney diseases [33], as confirmed by many studies [21]. As both the etiologic factor and the existing correlations with systemic diseases are known, removing the cause could lead not only to oral health improvement, but also to a reduction of the risk factors associated with these pathologies [34].

### 4.2. Alzheimer’s disease

Alzheimer’s disease is a neurodegenerative disease [35], which encompasses a heterogenous group of diseases characterized by a slow and progressive loss of one or more functions of the nervous system. The most well-known of these is Alzheimer’s disease, followed by Parkinson’s disease, lateral amyotrophic sclerosis, and Huntington’s chorea [36]. 

A recurrent sign of these pathologies is represented by dementia, defined as “a relevant or complete loss of intellectual capacities, beginning gradually and progressively, differentially caused in previously normal patients” [37].

Etiologically, neurodegenerative pathologies can be classified as primary or idiopathic degenerative dementia and dementia in the presence of other neurodegenerative pathologies. Epidemiologically, dementia affects 50% of elderly patients; half of this sample is related to Alzheimer’s disease. Its incidence tends to progressively increase in ≥65-year-old patients, reaching a prevalence of 4% in 75-year-old patients. For ≥75-year-old patients, new Alzheimer’s disease cases paradoxically tend to gradually decrease. As the average life expectancy has increased, it is clear how a disease mainly affecting 65-year-old to 75-year-old patients represents a remarkably relevant problem for public health [38].

In this systematic review, we will focus more on Alzheimer’s disease because it represents most primary neurodegenerative dementia cases. This pathology is characterized by a typical symptomatology constituted by an initial amnesiac deficit, followed by apraxia associated with space–visual and perceptive–visual disturbances, leading to, in its last stage, delirium and hallucinations. This symptomatology is correlated to histopathologic alterations in the cerebral cortex such as neurofibrillary tangles, senile plaques and amyloid plaques [39].

Age and heredity are the two key risk factors for Alzheimer’s disease onset. Regarding heredity, pathogenetic mutations of the amyloid precursor protein (APP) and of presenilin (I and II) determine a 50% increment of the onset risk [40]. There are different hypotheses about the etiopathogenesis of Alzheimer’s disease. The typical accepted hypothesis involves histopathologic alterations of senile plaques, neurofibrillary tangles, and neural cells caused by amyloid precipitation [41].

The expected models consider altered APP metabolism as a probable mechanism, resulting in an increased deposition of β-amyloid, a glycoprotein [42]. This can be due to either an increased or normal production of APP, but with a longer C-term chain that facilitates fibrillar deposition and formation. Other mutations implied in the etiopathogenesis can affect transmembrane proteins coded by the presenilin gene. β-amyloid deposition is neurotoxic, because it presumably causes an alteration in calcium homeostasis and in the production of free radicals. Although amyloid deposition is considered to play an important role in the etiopathogenesis, any aspects of cell death caused by it are still unknown.

These studies were performed on familiar types of Alzheimer’s disease in which the genetic causative agent could be more easily found. In classical forms of Alzheimer’s disease, the leading cause of amyloid deposition still has to be identified [43].

### 4.3. Inflammatory Theory

Besides the classic theory, an alternate theory claiming that β-amyloid precipitation could be caused by inflammatory processes also exists [44]. β-amyloid precipitation has been associated both with local sub-inflammatory contexts (CNS) and with peripherial inflammatory contexts, especially when stimulated by Gram-negative bacteria such as those representing the etiologic factors of periodontitis. 

The foundation of the inflammatory hypothesis related to Alzheimer’s disease is represented by a chronic self-triggered inflammatory process in the central nervous system, inducing neurodegeneration.

The factors causing and sustaining the inflammation through the course of Alzheimer’s disease are unknown, but some hints are given by studies conducted on senile plaques and neurofibrillary tangles. In fact, different factors have been discovered, like Aβ42 (beta-amyloid) [45] inside plaques and P-Tau (abnormal P-Tau phosphorylation causes damage to neuronal cytoskeleton) found in neurofibrillary tangles which, along with neuronal cell components facing apoptotic degeneration, are able to induce the synthesis of proinflammatory cytokines such as tumor necrosis factor (TNF-α), interleukin-1β (IL-1β), interleukin-6 (IL-6), and the production of CRP (C reactive protein) [46].

These factors not only sustain the inflammatory process, but are also able to induce neurodegeneration. Moreover, the elements cited above, along with proinflammatory cytokines, are able to active the complement chain, amplifying the conditions predisposing neurodegeneration. These hypotheses are being confirmed through in vitro, clinical, and epidemiological studies. In vitro studies have confirmed some points: TNF-α, IL-1β, and IL-6 have been shown to induce the synthesis of Aβ42 and P-Tau phosphorylation [47], which in turn induces the release of proinflammatory cytokines. Some clinical studies have found an increase of CRP whey levels (a protein involved in the acute phase of inflammation that increases during systemic inflammation) and other inflammatory markers in patients who have progressively developed Alzheimer’s disease [48]. 

Other studies have highlighted an increase in IL-6, IL-1β, and TNF-α associated with a greater incidence of intellectual ability loss in elderly patients [49]. Effectively, the role of proinflammatory cytokines as predictive factors for the development of Alzheimer’s disease are discordantly accepted by the scientific literature: these factors are implicated in the relationship to other systemic inflammatory diseases [50]. Therefore, there is an association, but certainly there is no specificity in the prediction of Alzheimer’s disease onset.

The inflammatory hypothesis is supported by studies about non-steroidal anti-inflammatory drugs (NSAIDs). Patients who were administered NSAIDs at the first signs of Alzheimer’s disease have shown a delay in the onset of dementia. On the other hand, other studies have shown a contradiction: the production of Aβ42 by neuronal glia, especially after the administration of indomethacin [51,52]. This contradiction could be caused by the fact that NSAIDs act through different mechanisms: on one hand, they increase the inhibition of COX-2 Aβ42 synthesis, but on the other hand, they can cause a reduction of proinflammatory cytokines such as those responsible for Aβ42 production [53,54].

Genetic studies about cytokines, specifically IL-α, have shown its notable genetic polymorphism and have found that patients expressing the IL-1α gene have a 10 times greater risk in developing Alzheimer’s disease [55]. Moreover, it should be noted that the presence of this gene is associated with a greater risk in developing periodontitis [56]. This study can point out the presence of other relationships between Alzheimer’s disease associations to neurodegeneration and periodontitis. 

Proinflammatory intermediates like TNF-α [22], IL-1, and IL-6 have limited access to the CNS as they are bulky [57]. There is evidence showing that these molecules reach or influence the CNS via the systemic circulation or neuronal paths. To reach the CNS, inflammatory cytokines have to cross the hematoencephalic barrier, which they can achieve in different ways such as modifying its permeability [58], binding to hematoencephalic barrier-free areas like circumentricular organs, passing through fenestrated capillaries, or using specific carriers [59].

Once they have reached the CNS, inflammatory cytokines can act to activate endothelial and perivascular cells located in the brain, inducing the synthesis of signaling molecules like nitrous oxide (NO), prostanoids, or other cytokines, which in turn induce glial cells. Naturally, if the neurodegeneration process has already started, these cytokines represent an additional trigger, amplifying inflammation and favoring the formation of pathological alterations [60].

Activated inflammatory cells (B and T lymphocytes and macrophages) can contribute to the inflammatory cerebral pool [61] by interacting with the CNS via neuronal pathways, interfering with peripheral nerve fibers (inducing them to synthesize proinflammatory cytokines) or going through these pathways to access the CNS [62]. This raises the possibility that peripheral inflammation could induce an increased synthesis of cytokines inside the CNS without a systemic increase. 

Bacterial products can increase cerebral cytokine levels. An example of this is bacterial lipopolysaccharide that is able to activate the innate immune system and induce the expression of CD14 on cell membranes, which in turn can be activated by β-amyloid [63]. Moreover, bacterial lipopolysaccharide can increase the permeability of the hematoencephalic barrier, allowing molecular and cellular components passage to the brain. This mechanism has been confirmed by a study performed on transgenic APPswe mice which were given bacterial lipopolysaccharide that lead to an increase in APP level [64].

Alzheimer’s disease pathogenesis involves different bacterial species, like *Chlamydia pneumoniae*, *Helicobacter pylori*, and spirochetes [58,65]. These bacteria have been found in different anatomical sections of the CNS, but their primary etiological role in the onset of Alzheimer’s disease is still being discussed, even if more evidence about their ability to invade the CNS and interfere with the basic mechanisms of the disease are coming to light.

### 4.4. Associative Hypotheses between Periodontitis and Neurodegenerative Diseases: Aspects in Common

Periodontitis and neurodegenerative diseases have a different etiopathogenesis, but they do share common risk factors that can influence their onset, gravity, and progression. The mechanisms involved in Alzheimer’s disease pathogenesis are not clear, but inflammation most likely plays a very important role; the inflammatory reactions increasing the cerebral inflammatory state can potentially favor the progression of the disease. Among these inflammatory reactions, periodontitis can be found, which exposes the host to the presence of a stable inflammatory process associated to bacteria colonizing the periodontium.

The proposed hypothesis is that periodontitis can in some way be involved in the natural history of Alzheimer’s disease [66]. Two periodontitis-associated mechanisms that could be related to Alzheimer’s disease are inflammation and bacteria [67,68].

The inflammatory mechanism involves the synthesis of inflammatory molecules caused by periodontitis, increasing the cerebral inflammation state. In fact, the interaction between periodontal bacteria and the host causes a local production of inflammatory molecules such as IL-1β, IL-6, IL-8, TNF-α, and CRP [69]. In the worst cases of periodontitis, proinflammatory cytokines can induce a systemic inflammation, which is potentially capable of reaching the central nervous system via systemic circulation [70]. These molecules produced in the periodontium can also trigger the trigeminal nerve in the oral cavity, further increasing the production of cerebral cytokines. Cytokines can have a synergic effect towards activated glia, causing an amplified reaction favoring Alzheimer’s disease progression.

It still unknown whether peripheric inflammation is involved in disease onset or in its progression, or both. It is presumed that bacteria involved in periodontitis pathogenesis could also be involved in Alzheimer’s disease pathogenesis. Species with a greater association to both pathologies include *A. actinomycetemcomitans*, *P. gingivalis*, *T. denticola*, and *F. nucleatum*. These bacteria can invade the CNS, triggering neurodegenerative disease by interacting with preexisting pathological mechanisms. Some studies have highlighted the presence of some species of *T. denticola* in the brain, both in living animals and in tissue sections. This validation should not be surprising because, as we know *T. denticola* is part of the same taxonomic genre as *Treponema pallidum* (the etiologic factor of syphilis), which has the ability to invade the CNS and to cause the deposition of amyloid. Also, the presence of the above-mentioned bacteria in cerebral abscesses provides proof of their ability to invade the brain.

Periodontal bacteria, with their virulence factors such as lipopolysaccharide, are able to induce cytokine release when they reach the CNS. This hypothesis has been confirmed by many studies; in fact, a worsening of the disease in mice affected by demyelinating encephalitis after the inoculation of heat-inactivated bacteria (*Porphiromonas gingivalis)* has been shown [71]. The main factor in stimulating glial cells, as previously described, is the lipopolysaccharide, especially the one associated to *P. gingivalis*. This glycoprotein has the ability to induce the release, from the glia, of both nitrous oxide and prostaglandin E2, as highlighted by studies performed on mice. This induction is mediated by the presence of receptors like CD14, TLR-2, and 4. An experiment performed on mice involving the subcutaneous injection of *C. rectus* [72] has underlined the presence of macroscopic anatomical alterations in the hippocampus region, cytoplasmic alterations like the presence of vacuoles and cellular residues and double the quantity of both TNF-α and interferon-γ in the progeny of test animals [73].

The mechanisms allowing periodontal bacteria to access the CNS still remain unknown, but it can be hypothesized that they use a similar pathway as other bacteria (via systemic circulation). This can cause bacteremia, which is most often a result of trauma or dental practice. Bacteria can also reach the CNS via peripheral nerves, following their path. In fact, various studies have highlighted the presence of spirochetes in trigeminal ganglion, proving that they can travel up peripheric nerves, invading the CNS [74]. Naturally, the presence of these bacteria in peripheral nervous fibers or in the systemic circulation does not imply that these bacteria can automatically access the CNS. Furthermore, it is hypothesized that the presence of different cofactors, like proinflammatory cytokines, other infections, and the patient’s age, contribute to how periodontal bacteria access the central nervous system.

Until now, clinical evidence showing a direct connection between periodontitis and Alzheimer’s disease has not been obtained. Nevertheless, there is indirect evidence. Given that tooth loss can be related to multiple causes, including periodontitis, it has been noticed that tooth loss is associated both with dementia and Alzheimer’s disease [75]. This evidence has been found by studies performed on two different population samples [76,77], showing a greater incidence of tooth loss associated with cognitive decline.

A Swedish study has proved the presence of a strong relationship between tooth loss and Alzheimer’s disease in homozygotic twins [78]. The Nun Study showed an increased risk in developing Alzheimer’s disease in patients with a reduced number of teeth, which was up to 6.4 times more compared to the general population. This risk increment is present in patients who do not have the APOE4ε allele. This study relates the absence of this allele to the concentration of IgG against periodontal bacteria.

The authors of the Nun Study have hypothesized that the presence of APOE4ε represents a risk factor for Alzheimer’s disease. Nevertheless, this increase in incidence can be related to a greater susceptibility of patients without the APOE4ε allele, and that actually represents the real risk factor. It has been hypothesized that the presence of this allele can positively or negatively influence the inflammatory reaction against the infection. Therefore, patients without the APOE4ε allele have better immunity against periodontal bacteria compared to patients with the APOE4ε allele, and such immunity represents the cause of a higher Alzheimer’s disease incidence in this sample. Naturally, tooth loss can be not only related to periodontitis, but also to cavities and to endodontic pathologies [78] and evidence associating these pathologies to Alzheimer’s disease may be forthcoming.

### 4.5. Analysis of the Evidence on the Role of P. gingivalis on the Etiopathogenesis of A.D.

The main associations between *P. gingivalis* and the onset of Alzheimer’s disease come from studies examining the inflammatory action of Porphyromonas against microglia, which is mediated by its LPS.

In fact, a 2017 study conducted by Wu et al. [9] found a strong association between LPS-pg administered to CatB-dependent mice and a reduction in learning and memory; the mechanism of action was mediated by the role of cathepsin B, which promotes the production of IL-1β and the production of β-amyloid from microglial cells. Wu et al. identifies a random association between chronic exposure to periodontal bacteria in middle-aged mice with a similar AD phenotype and learning and memory impairment. Chronic systemic exposure with LPS-pg is conducted for five weeks, inducing a memory deficit in middle-aged mice but not in young mice. In addition, the exposure sustained by LPS-pg mice did not reduce body weight or induce lethargy, suggesting that the reduction of learning and memory are not associated with disease behavior in mice. There was no LPS-pg-induced memory deficit in CatB^−/−^ mice, but there was a significant increase in CatB in the microglia and neurons in CatB-dependent mice, suggesting that the memory deficit is due to systemic exposure of LPS-pg in middle-aged CatB-depedent mice [9].

CatB plays an important role both in the mediation of neuroinflammation (TLR expression increase) and β amyloid deposition. Wu therefore identifies CatB as a therapeutic target for its role in the connection between periodontal disease and the onset and progression of AD [9]. Cathepsin b is also identified as a therapeutic target in a study on mice in 2019 by Nie et al. [18].

Furthermore, Nie shows that infection of mice with *P. gingivalis* induces the production of beta amyloid through the activation of CatB/NF-κB and the increase in the expression of TLR-2 and IL-1b [18].

These data are partly confirmed in a study by Hayashi et al. in 2019 [10], who found a worsening of the ability to learn and memorize in middle age mice exposed to LPS-pg, and in a study by Liu et al. in 2017 [13].

Hayashi found that the continuous injection of LPS-pg induces not only sarcopenia and cardiac lesions in young and middle-aged AD mice, but also poor results in learning scores. Contrary to previous studies, Hayashi does not identify an increase in the expression of TLR 2, TLR 4, NF-κB, and Cox-2, indicating that a weak inflammatory response from LPS-pg could not modulate an inflammatory response mediated by TLR [10].

Liu instead sought to investigate the role of gingipain, which includes Arg-gingipain (Rgp) and Lys-gingipain (Kgp). These two molecules are a class of cysteine proteinases produced by *Porphyromonas*, providing the first evidence that these two proteases contribute cooperatively to the cell migration induced by *P. gingivalis* and the expression of proinflammatory mediators through the activation of the protease-activated receptor (PAR) 2 [13].

The role of LPS-pg in AD etiopathogenesis is also confirmed in a study conducted on brain tissues (post mortem) of patients with Alzheimer’s disease by Poole et al. in 2013 [11].

Poole investigated the presence of three periodontal pathogenic bacteria (*P. gingivalis*, *T. denticola*, and *T. forsythia*) in sections of brain tissue of patients with AD. Only the presence of Porphyromonas on examination by indirect immunofluorescence was found. Furthermore, Poole gave further evidence by demonstrating that neuronal glia cells (SVGp1) adsorb LPS from a culture of *P. gingivalis*, which also contained extracellular cysteine proteases (gingipain) and metabolites such as butyric and propionic acids in addition to LPS. Of these, LPS was adsorbed on the surface of the membrane of an astroglial cell line, whereas gingipain demonstrated intracellular localization [11].

The presence of proinflammatory cytokines, such as IL-1β, TNF-α, and CRP, together with the presence of β-amyloid were found in the brain tissues of mice where periodontitis was induced by the inoculation of *P. gingivalis* in the periodontal tissue [16].

A recent study carried out on mice and CSF fluid from a patient with AD conducted by Dominy has confirmed the main suspect to be *P. gingivalis* and subsequently gingivitis in AD etiopathogenesis [17]. The results of this study offer evidence that *P. gingivalis* and gingipain play a central role in the pathogenesis of AD. The presence of *P. gingivalis* DNA and gingival antigens in AD brains has been shown, and oral administration of small molecule gingipain inhibitors blocks induced gingipain neurodegeneration, significantly reducing the load of *P. gingivalis* in the mouse brain and decreasing the response to *P. gingivalis* brain infection.

In 2017, a pilot study on patients with dementia and Alzheimer’s disease investigating the presence of antibodies in serum and CSF (against *P. gingivalis*, *T. forsythia*, *T. denticola*, *T. socranskii*, and *A. actinomycetemcom*) found no statistically significant difference between the two groups [14]. This study does not confirm the hypothesis of microorganisms playing a role in the progression and onset of AD pathology [75].

Other clinical studies (an observational study [22], case–cohort study [20], observational cohort study [15], longitudinal study [19], and prospective pilot study [17]) that investigated the presence of antibodies against *P. gingivalis* agree on the role of *P. gingivalis* in the onset and progression of Alzheimer’s disease, placing it as a potential risk factor.

Noble et al. observed an increased risk of incidence of AD among participants with elevated serum anti-*A. naeslundii* IgG antibody in a cohort study. In addition, elevated serum levels of *E. nodatum* IgG have been associated with a low risk of AD. *E. nodatum* is associated with aggressive periodontitis, whereas *A. naeslundii* is involved in dental plaque formation, gingivitis, and dental caries [20].

In a six-month observational cohort clinical study, Ide et al. investigate the presence of periodontitis, serological markers, and inflammatory cytokines in a population of 60 patients with mild to moderate AD. *P. gingivalis* IgG concentration, TNF-α, and IL-10 correlated with the progress of cognitive decline. In this study, Ide et al. does not find a correlation between serum levels of *P. gingivalis* IgG and cognitive decline but only a correlation between periodontal disease and AD [15].

Stein et al. investigated the serological antibody levels of seven periodontal pathogens (Aggregatibacter actinomycetemcomitans, Porphyromonas gingivalis, Campylobacter rectus, Treponema denticola, Fusobacterium nucleatum, Tannerella forsythia, and Prevotella intermedia) in 158 subjects in a longitudinal study. Out of 158 participants, 81 developed cognitive decline and 77 remained cognitively intact. Antibody levels were compared between the two groups. The results show that in patients who have undergone AD, there is a high antibody response towards F. nucleatum and P. intermedia compared to patients without Alzheimer’s. This study provides initial data demonstrating high antibody responses against the bacteria involved in periodontal disease in subject’s years before cognitive impairment, suggesting that periodontal disease could potentially contribute to the risk of AD onset/progression [15].

Two bioinformatics studies investigated 78 genes associated with Alzheimer’s disease by relating them to different pathogens (including *P. gingivalis*) and the immune system, confirming possible associations between periodontal disease caused by *P. gingivalis* and AD, concluding that it is therefore not unreasonable to suggest that antibiotics, antifungal, and antiviral agents, possibly in combination and adapted to the individual, may be able to arrest, delay, or even provide remission in patients with AD [12].

### 4.6. Health and Oral Hygiene in Patients with Alzheimer’s Disease

A recent review conducted by Orr et al. in 2019 brings to the fore the aspect of oral health and hygiene in patients suffering from dementia with cognitive decline, with the aim of identifying predictive factors of the progression of the disease. The review highlights how, following the decline of cognitive functions, oral health, and specifically oral hygiene tends to decrease, along with decreasing access to dental care [79].

Although the decline in oral hygiene follows cognitive decline, oral health with the loss of dental elements at a young age could be interpreted as a possible predictor of dementia.

Evidence of direct involvement of oral health and oral hygiene in the etiopathogenesis of Alzheimer’s disease comes from several studies. In fact, in a study by Kobayashi et al. [80], the loss of dental elements in patients who are not demented was associated with the atrophy of gray matter in the hippocampus, the caudate nucleus, and the right temporal pole (areas dedicated to learning and memorization).

Takagi et al. identify a reduction in swallowing in Alzheimer’s patients with reduced ability to ingest and digest food which affects oral and systemic health [81].

Studies conducted on animal models have shown a reduction in learning and memory in mice where, after dental eruption or in adulthood, the teeth are extracted, or in mice that have had a diet with soft foods [82].

In these studies, we can see how the loss of sensory input from the teeth, and not the physical loss of the teeth, affects spatial learning and memory in rodents.

In a recent systematic review, Delwen argues that older people with dementia have high plaque levels and oral health problems related to oral soft tissues, such as gingival bleeding, periodontal pockets, stomatitis, mucosal lesions, and reduced salivary flow [83].

Certainly with the onset of Alzheimer’s disease, the patient’s ability to comply with oral hygiene maneuvers tends to decrease. Oral health problems consequently worsen both for soft and hard tissues, but also the loss of hard oral tissues such as teeth at an early age can directly and negatively affect the onset and progression of Alzheimer’s disease without local oral inflammatory mechanisms in place.

Oral hygiene and oral health of older people with dementia should be improved. This could be achieved through oral hygiene education of assistants assigned to the care of the elderly and to carrying out controlled visits in order to monitor the oral health of the patient.

## 5. Conclusions

Alzheimer’s disease and its consequent cognitive decline represent a severe public health problem, considering the increase of its incidence, the prolongation of the average life expectancy, and its great impact on quality of life. Knowledge of its pathophysiological mechanisms is not sufficient yet, so the preventive approach related to known and/or unknown risk factors could mean a more efficient management of the patient and consequently a better quality of life. Therefore, the present paper aims to evaluate whether there are any aspects of periodontitis that could potentially relate to neurodegenerative disease pathogenesis, thus representing a risk factor; this could be relevant because nowadays, periodontitis is fully treatable and preventable. This literature review evaluated the contribution of periodontitis to peripheral inflammation through a direct effect by periodontal pathological bacteria [84] or through an indirect effect caused by proinflammatory cytokines; this contribution could have a role as a cofactor to induce or accelerate the progress of AD thanks to the most recent etiopathogenetic theories giving inflammatory mechanisms a key role for this pathology. Analyzing the characteristics of both pathologies, we focused on their common aspects: high prevalence, peak of incidence, inflammatory pathogenesis, etc.

The literature proposes the hypothesis that the bacterial load and the inflammatory reaction related to periodontitis can intensify the inflammation in the central nervous system, eventually favoring the onset of disease [7,85]. Even if no direct evidence associates periodontitis with Alzheimer’s disease, preliminary indirect evidence is provided by a few clinical and epidemiological studies available. Particularly, the Nun Study analysis proposed a small number of teeth to be associated with the presence of dementia in old age [76]; however, it was not able to establish if this association is completely casual or partially casual. Moreover, this study evaluated the role of the APOE4ε; its absence would predispose to Alzheimer’s disease, but the mechanism behind this was not explained. 

The study performed using the third National Health and Nutrition Examination Survey (NHANES III) derived data has related the presence of high IgG levels against *P. gingivalis* to a greater possibility of memory and cognitive impairment [86]. In fact, a statistically significant relationship between IgG, *P. gingivalis* and dementia has been found. From these studies, a correlation between periodontitis and neurodegenerative diseases can be deduced, but neither can definitely prove their possible association nor demonstrate a possible mechanism through which periodontitis can induce the onset or the evolution of neurodegenerative diseases [87]. 

However, if these hypotheses were validated by future studies, the relevance of such gain would have immediate and significative implications. Particularly, the prevention and treatment of periodontitis could reduce the patient’s risk profile towards the development of Alzheimer’s disease [88]; there will be evidence that peripheral infections would need greater attention. In conclusion, today’s evidence seems to propose a possible relationship between periodontitis and neurodegenerative diseases, especially Alzheimer’s disease [89].

Nevertheless, today there is no definitive evidence to consider periodontitis as a risk factor, so more research will have to be conducted on this topic. Future studies that correlate periodontitis and Alzheimer’s disease should mainly focus on the possibility of preventing the onset of periodontal disease, the loss of dental elements, the reduction of local inflammation, and the predisposing oral factors of AD. Furthermore, the role of periodontal bacteria in the etiopathogenesis of Alzheimer’s disease should be investigated.

## Figures and Tables

**Table 1:** Complete overview of the search methodology. Records identified by databases: 1088; records selected for qualitative analysis: 36.

Database—Provider	Key Words	Search Details	Number of Records	Number of Records) after Limiting by Year of Publication (last 30 years)	Number of Studies Dealing with the Topic of Alzheimer’s Disease in Relation to Oral Inflammatory Processes and Bacteria	Number of Articles Investigating the Role of Periodontal Bacteria in the Onset and Progression of Alzheimer’s Disease	Articles after Removing Overlaps	Number of Articles Included in the Qualitative Analysis
PubMed	“Alzheimer’s Disease” AND “periodontal”	“Alzheimer’s Disease” (All Fields) AND “periodontal” (All Fields)	96	96	48	7		
PubMed	“Alzheimer’s Disease” AND “periodontitis”	“Alzheimer’s Disease” (All Fields) AND “periodontitis” (All Fields]	87	87	56	9		
PubMed	“dementia “AND “periodontal”	“dementia “(All Fields] AND “periodontal” (All Fields)	118	117	41	3		
PubMed	“Alzheimer’s Disease” AND “actinomycetemcomitans”	“Alzheimer’s Disease” (All Fields] AND “actinomycetemcomitans” (All Fields)	3	3	3	2		
PubMed	“Alzheimer’s Disease” AND “gingivalis “	“Alzheimer’s Disease” (All Fields) AND “gingivalis” (All Fields)	34	34	20	20		
PubMed	“Alzheimer’s Disease” AND “denticola “	“Alzheimer’s Disease” (All Fields) AND “denticola” (All Fields)	9	9	7	6		
PubMed	“Alzheimer’s Disease” AND “nucleatum”	“Alzheimer’s Disease” (All Fields) AND “nucleatum” (All Fields)	4	4	4	3		
Scopus	“Alzheimer’s Disease” AND “periodontal“	TITLE-ABS-KEY (“Alzheimer’s Disease” AND “periodontal”)	234	232	84	8		
Scopus	“Alzheimer’s Disease” AND “periodontitis”	TITLE-ABS-KEY (“Alzheimer’s Disease” AND “periodontitis”)	182	182	48	11		
Scopus	“dementia “AND “periodontal”	TITLE-ABS-KEY (“dementia” AND “periodontal”)	217	214	71	4		
Scopus	“Alzheimer’s Disease” AND “actinomycetemcomitans”	TITLE-ABS-KEY (“Alzheimer’s Disease” AND “actinomycetemcomitans”)	8	8	7	7		
Scopus	“Alzheimer’s Disease” AND “gingivalis “	TITLE-ABS-KEY (“Alzheimer’s Disease” AND “gingivalis “)	64	64	31	19		
Scopus	“Alzheimer’s Disease” AND “denticola “	TITLE-ABS-KEY (“Alzheimer’s Disease” AND “denticola”)	20	20	16	9		
Scopus	“Alzheimer’s Disease” AND “nucleatum”	TITLE-ABS-KEY (“Alzheimer’s Disease” AND “nucleatum”)	12	12	8	6		
Total records			1088	1082	444	114	36	15

**Table 2:** K agreement calculation. Proportion of agreement (Po) = 0.855; agreement expected (Pe) = 0.4105; K agreement = 0.754. No agreement: 0.0–0.20; slight agreement: 0.21–0.40; fair agreement: 0.41–0.60; moderate agreement: 0.61–0.80; substantial agreement: 0.81–1.00). The K agreement was calculated from 36 articles to include 15 articles with the application of the inclusion and exclusion criteria.

		Reviewer 2	Reviewer 2	Reviewer 2	
		Include	Exclude	Unsure	Total
Reviewer 1	include	15	2	1	18
Reviewer 1	exclude	0	13	0	13
Reviewer 1	unsure	1	3	1	5
	total	16	18	2	36

**Table 3:** Extracted data for secondary outcome.

Author, Data, and Journal	Type of Study	Cell Lines, Tissue, Animals, and Databases	Antibodies, Antigens, Enzyme, and Proteins Investigated	Investigated Microorganisms	Results
Wu et al., 2017, Brain Behav Immun [9]	Experimental study on mice and cell lines	(CatB^−/−^) mice, MG6 microglia cell line,	Pg- LPS, CatB	*P. gingivalis*	CatB plays a critical role in the link between periodontitis and AD.
Hayashi et al. 2019, Exp Gerontol [10]	Experimental study on mice	Mice	Pg- LPS	*P. gingivalis*	LPS pg exposure worsens the prognosis in AD
Poole rt al. 2013, J Alzheimers Dis [11]	Experimental study on brain tissue and cell lines	Postmortem brain tissue, SVGp12 cells	LPS	*T. denticola* *T. forsythia* *P. gingivalis*	Associative hypothesis between LPS pg and AD
Carter et al 2017, Journal of Alzheimer’s disease reports [12]	Bioinformatics study on databases	GWAS databases	78 AD genes (GWAS)	*Bacteria*, *viruses*, *fungi*	The use of antibiotics and antifungals could reduce the effects of AD
Liu et al. 2017, Sci Rep [13]	Experimental study on mice and cell lines	Mice, MG6 microglia cell line	Arg-gingipain (Rgp) and Lys-gingipain (Kgp)	*P. gingivalis*	Data supports the infection hypothesis of Alzheimer’s disease
Laugish et al.2018, J Alzheimers Dis [14]	Clinical study	Patients with dementia (N = 20 in AD and N = 20 dementia non AD)	Tau protein (T-tau) and Amyloid β (Aβ1-42) in CFS(Cerebro spinal fluid) and Antibody levels in CFS e serum	*P. gingivalis* *T. forsythia* *T. denticola* *T. socranskii* *A.actinomycetemcom*	The data does not support an associative hypothesis
Ide et al. 2016, PLoS One [15]	Observational cohort study	Patients with dementia (*n* = 60)	Serum inflammation, antibody and DNA assays	*P.gingivalis*	Study suggests there is a direct relationship between periodontitis and cognitive decline
Ishida et al. 2017, NPJ aging and mechanisms of disease [16]	Experimental study on mice	Mice	Amiloid β (Aβ) deposition, Aβ40, Aβ42, IL-1β and TNF-α	*P. gingivalis*	Concludes that periodontitis is truly a risk factor for AD
Dominy et al. 2019, Sci Adv [17]	Prospective pilot study	Mice and AD patients.	(Kgp), (Rgp), *P. gingivalis* 16S rRNA gene, P. gingivalis DNA in CFS, Aβ1–42 in cerebral mice tissue	*P. gingivalis*	*P. gingivalis* and gingipains in the brain play a central role in the pathogenesis of AD
Nie et al. 2019, J Alzheimers Dis [18]	Experimental study on mice	Mice	IL-1β, AβPP770, CatB, Aβ1-42, and Aβ3-42 in macrophage/monocites	*P. gingivalis*	Taken together, CatB may be a novel therapeutic target for preventing the periodontitis-related AD initiation and pathological progression.
Diaz-Zuniga et al. 2019, J Oral Microbiol [6]	Experimental study on cell line rat	Line rat cell (Mixed hippocampal cultures, Microglial cultures)	IL-1β, IL-6, TNF-α and Aβ1-42	*A. actinomycetemcomitans*	Probable association between aparodontal disease sustained by *Aggregatibacter* and AD etiopathology
Sparks Stein et al. 2012, Alzheimer’s Dement [19]	Longitudinal study	Patient AD = 35, MCI = 46 and control = 76	Antibody levels	*A. actinomycetemcomitans P. gingivalis*, *C. rectus*, *T. denticola, Fusobacterium nucleatum*, *T. forsythia*	Possible association between antibody levels and onset and progression of AD
Noble et al. 2014, PLoS One [20]	Case–cohort study	Patients who developed AD in follow-up	Serum IgG	*P. gingivalis*, *T. forsythia A. actinomycetemcomitans T. denticola*, *C. rectus*, *E. nodatum* and *A. naeslundii*	Serum IgG levels to common periodontal microbiota are associated with risk for developing incident AD
Carter et al. 2017, Front Aging Neurosci [21]	Bioinformatics study on databases	GWAS databases	P. gingivalis/host interactome	*P. gingivalis*	Supports the many documented relationships between *P. gingivalis* infection and AD or its comorbid conditions
Kamer et al. 2009, J Neuroimmunol [22]	Observational Study	18 with AD and 16 cognitively normal	Plasma TNF-α, IL-1β and IL-6 levels, IgG antibody	*A. actinomycetemcomitans serotype b*, *T. forsythia* and *P. gingivalis*	Antibody levels to periodontal bacteria associate with AD and may help improve the clinical diagnosis of AD
